# Supplementary material for: A novel ENU-induced ankyrin-1 mutation impairs parasite invasion and increases erythrocyte clearance during malaria infection in mice
Source: Sci Rep. 2016 Nov 16;6:37197. doi: 10.1038/srep37197 (PMC5111128; doi:10.1038/srep37197)
Supplement: Supplementary Information [file srep37197-s1.pdf]

A novel ENU-induced ankyrin-1 mutation impairs parasite invasion and increases erythrocyte clearance during malaria infection in mice

Hong Ming Huang[1], Denis C. Bauer[2], Patrick M. Lelliott[3], Andreas Greth[4], Brendan J. McMorran[1], Simon J. Foote[1], Gaetan Burgio[1*]

[1] Department of Immunology and Infectious Disease, John Curtin School of Medical Research, Australian National University, ACT, Australia.

[2] CSIRO, Sydney, NSW, Australia.

[3] IFReC Research Building, Osaka University, 3-1 Yamada-oka, Suita, Osaka 565-0871, Japan.

[4] synaps studios GmbH, Rebmoosweg 73A, CH-5200 Brugg, Switzerland.

***Correspondence to:** Dr. Gaetan Burgio**:** The John Curtin School of Medical Research, Australian National University,131 Garran Road, ACT 2601, Australia. email: Gaetan.burgio@anu.edu.au

Supplementary figure 1

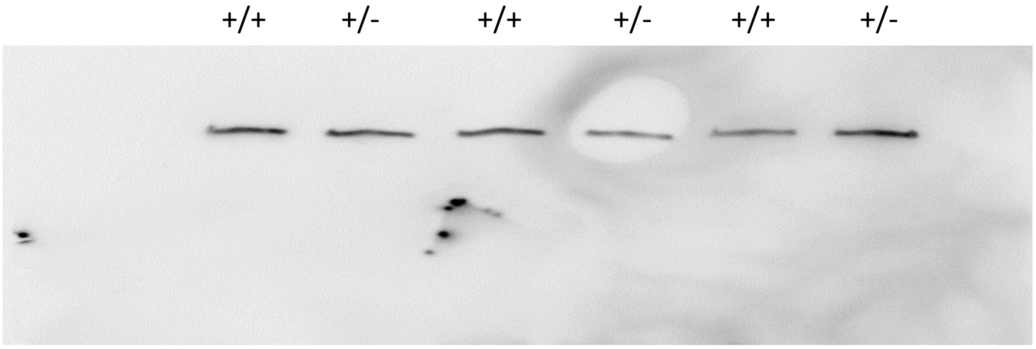

Supplementary figure 1. **The full western blot membrane when probed with anti-beta-spectrin antibody.** The disparity of band intensity as shown in Figure 3c is due to uneven surface of the membrane rather than post-processing issue.

Supplementary figure 2

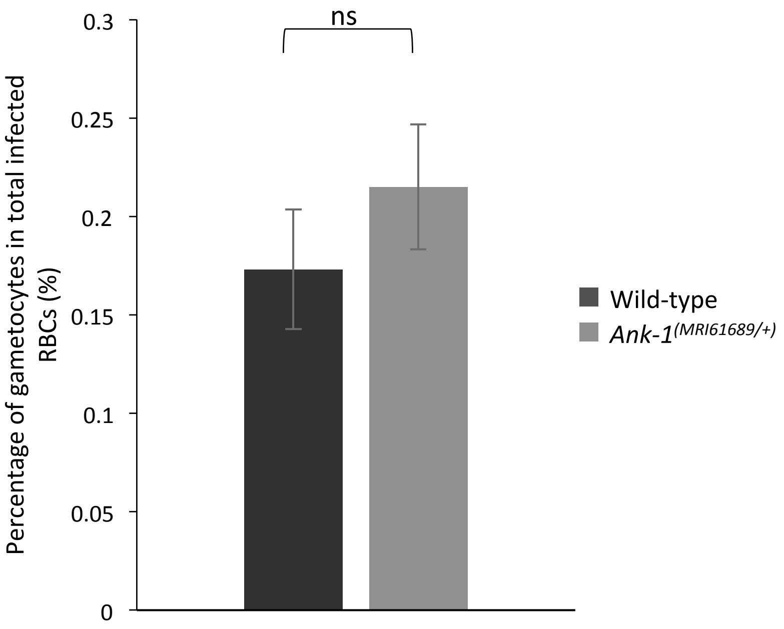

Supplementary figure 2. **The percentage of gametocytes of wild-type and of *Ank-1*$^{(MRI61689/+)}$ mice during malaria infection.** Parasite gametocyte numbers were counted under light microscopy at 15-30% parasitaemia, and the proportion of gametocytes to total infected RBCs were calculated (n=6). Error bars indicate SEM.

Supplementary figure 3

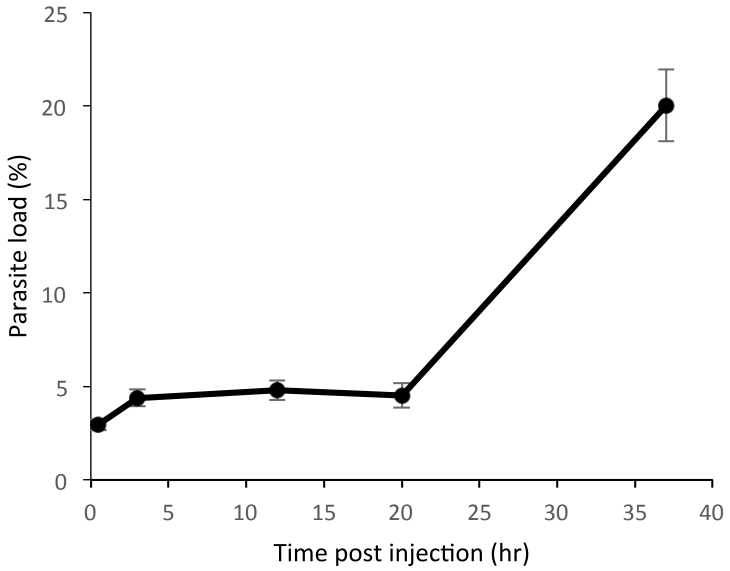

Supplementary figure 3. **The parasite load of the mice during IVET assays.** The parasite load of the host mice during IVET assay (n=7). Error bars indicate SEM.
